# VSV-ΔG-Spike Candidate Vaccine Induces Protective Immunity and Protects K18-hACE2 Mice against SARS-CoV-2 Variants

**DOI:** 10.3390/v15061364

**Published:** 2023-06-13

**Authors:** Yfat Yahalom-Ronen, Hadas Tamir, Sharon Melamed, Boaz Politi, Hagit Achdout, Noam Erez, Ofir Israeli, Inbar Cohen-Gihon, Lilach Chery Mimran, Moria Barlev-Gross, Michal Mandelboim, Irit Orr, Ester Feldmesser, Shay Weiss, Adi Beth-Din, Nir Paran, Tomer Israely

**Affiliations:** 1 Department of Infectious Diseases, Israel Institute for Biological Research, Ness Ziona 74100, Israel; yfatyr@iibr.gov.il (Y.Y.-R.); hadast@iibr.gov.il (H.T.); sharonm@iibr.gov.il (S.M.); boazp@iibr.gov.il (B.P.); hagita@iibr.gov.il (H.A.); noame@iibr.gov.il (N.E.); lilachc@iibr.gov.il (L.C.M.); moriab@iibr.gov.il (M.B.-G.); shayw@iibr.gov.il (S.W.); 2 Department of Biochemistry and Molecular Genetics, Israel Institute for Biological Research, Ness Ziona 74100, Israel; ofiri@iibr.gov.il (O.I.); inbarg@iibr.gov.il (I.C.-G.); adib@iibr.gov.il (A.B.-D.); 3 Central Virology Laboratory, Ministry of Health, Sheba Medical Center, Tel Hashomer, Ramat Gan 76100, Israel; michal.mandelboim@sheba.health.gov.il; 4 Bioinformatics Unit, Life Science Core Facilities, Weizmann Institute of Science, Rehovot 52621, Israel; irit.orr@weizmann.ac.il (I.O.); ester.feldmesser@weizmann.ac.il (E.F.)

**Keywords:** SARS-CoV-2, VSV-ΔG-Spike, vaccine, variants, K18-hACE2, brain

## Abstract

Since the emergence of the original SARS-CoV-2, several variants were described, raising questions as to the ability of recently developed vaccine platforms to induce immunity and provide protection against these variants. Here, we utilized the K18-hACE2 mouse model to show that VSV-ΔG-spike vaccination provides protection against several SARS-CoV-2 variants: alpha, beta, gamma, and delta. We show an overall robust immune response, regardless of variant identity, leading to reduction in viral load in target organs, prevention of morbidity and mortality, as well as prevention of severe brain immune response, which follows infection with various variants. Additionally, we provide a comprehensive comparison of the brain transcriptomic profile in response to infection with different variants of SARS-CoV-2 and show how vaccination prevents these disease manifestations. Taken together, these results highlight the robust VSV-ΔG-spike protective response against diverse SARS-CoV-2 variants, as well as its promising potential against newly arising variants.

## 1. Introduction

The ongoing coronavirus disease 2019 (COVID-19) pandemic, caused by severe acute respiratory syndrome coronavirus 2 (SARS-CoV-2), presents a global threat to public health, and it is still taking its toll, with over 750 M cases worldwide, as well as over 6.8 M deaths (As of 12 April 2023). Along with the pandemic, variants emerged, including alpha, which first appeared in the United Kingdom (UK or B.1.1.7), beta, originally described in South Africa (SA or B.1.351), gamma, identified in Brazil (P.1), delta, which emerged in India (21A/S:478K or B.1.617.2), and omicron (B.1.1.529) (https://www.cdc.gov/coronavirus/2019-ncov/variants/variant-info.html (accessed on 11 June 2023)). These variants show increased transmissibility (largely alpha, delta, and omicron), immune evasion (primarily beta and gamma, but also delta and omicron), or both (mostly delta and omicron) [1,2,3,4,5]. The ongoing evolution of SARS-CoV-2 and the consequential emergence of various SARS-CoV-2 variants, as well as subvariants, such as “Omicron variants under monitoring”, as recently defined by the WHO (https://www.who.int/activities/tracking-SARS-CoV-2-variants, updated on 18 January 2023), which vary in their sequence, transmissibility, the nature of the disease they cause, challenge the efficacy of currently available vaccines and therapeutic solutions.

VSV-ΔG-spike is a replication-competent vaccine candidate, based on the Vesicular Stomatitis Virus (VSV) platform, in which the VSV glycoprotein G (VSV-G) was replaced by a full-length human codon-optimized Spike glycoprotein (S) gene of SARS-CoV-2. [6]. VSV-ΔG-spike propagation was performed by serial passaging in vero E6 and vero cells, a process that was accompanied by spontaneous accumulation of several mutations, until reaching a stable genetic version of the vaccine [6,7]. These abovementioned mutations include known spike mutations at N501, E484, Q493, and G685, sites of major importance to immune escape and virus transmissibility, as previously described [8,9].

Previously, we showed that sera derived from individuals participating in phase II clinical trial vaccinated with VSV-ΔG-spike efficiently neutralized the original virus, as well as alpha, beta, gamma, and delta [7]. This observed preservation may be attributed to the unique sequence composed of spike mutations that correspond with SARS-CoV-2 variants’ mutations, which occurred naturally during growth and evolution of the VSV-ΔG-spike vaccine [7].

In the current work, we aimed to explore the robustness of the VSV-ΔG-spike vaccine candidate against various SARS-CoV-2 variants. To that end, VSV-ΔG-spike efficacy was evaluated against authentic SARS-CoV-2 variants: alpha, beta, gamma, and delta, in comparison to the original virus, in K18-hACE2 mice. K18-hACE2s are transgenic mice designed to express the human ACE2 receptor under the keratin 18 promoter (K18) in epithelial cells in various tissues, including airway epithelia, namely, the route of SARS-CoV-2 infection, and they are widely used as SARS-CoV-2 infection models [10]. It was previously reported that SARS-CoV-2 infection of K18-hACE2 mice targets the brain as a major site, leading to severe neurological disease, neuroinflammation, and neuronal death, contributing to infection-associated morbidity and mortality [11,12].

Here, we tested the efficiency of the VSV-ΔG-spike candidate vaccine against authentic SARS-CoV-2 viruses. We show that VSV-ΔG-spike vaccination induces neutralizing antibodies against all tested variants (original virus, alpha, beta, gamma, and delta), and it provides protection against lethal doses of each variant. Moreover, we show a significant prevention of viral load in organs of vaccinated mice, as well as a reduction in lung and brain tissue damage, compared to unvaccinated mice infected with each of the variants. RNAseq analysis of infected brains revealed that, whereas infection with any of the tested SARS-CoV-2 variants led to induction of cytokine storm and extensive neuroinflammation response, vaccination prior to infection completely prevented these deleterious phenotypes. We previously showed the efficacy of the VSV-ΔG-spike vaccine in serum samples derived from vaccinees participating in phase II clinical trial of VSV-ΔG-spike vaccine against variants alpha, beta, gamma, delta, and omicron [7]. Thus, though not tested in this current work, we suggest that VSV-ΔG-spike may prove efficacious against the currently predominant variant omicron and its subvariants.

Taken together, we show that disease outcome and harmful phenotype caused by lethal dose of the original virus or variants alpha, beta, gamma, or delta can be prevented by VSV-ΔG-spike vaccination, irrespective of variant identity.

## 2. Methods

### 2.1. Cells

Vero E6 cells (ATCC CRL-1586™) were grown in DMEM containing 10% fetal bovine serum (FBS), MEM nonessential amino acids (NEAA), 2 mM L-glutamine, 100 Units/mL penicillin, 0.1 mg/mL streptomycin, and 12.5 Units/mL nystatin (P/S/N). Calu3 cells (ATCC HTB-55) were grown in RPMI, supplemented with 10% FBS, NEAA, 2 mM L-glutamine, P/S/N, and 1% Na-pyruvate. All reagents were from Biological Industries, Beit-Haemek, Israel. Cells were cultured at 37 °C, being in conditions of 5% CO_2_ with 95% humidity.

### 2.2. Viruses

Virus stocks of the SARS-CoV-2 original virus (GISAID accession EPI_ISL_406862) were propagated (four passages) in Vero E6 cells. SARS-CoV-2 variants were provided by the Central Virology Lab of the Israel Ministry of Health [13]. Alpha (B.1.1.7, GISAID accession EPI_ISL_4169857) was passaged once in Vero E6, followed by two passages in Calu3 cells. Beta (B.1.351, GISAID accession EPI_ISL_4169885), gamma (P.1, GISAID accession EPI_ISL_4169886), and delta (B.1.617.2, GISAID accession EPI_ISL_4169986) variants were propagated in Vero E6 cells. Variants were verified by whole-genome sequencing (WGS) [7]. All virus stocks were titered on Vero E6 cells, as previously described [6]. Handling and working with the SARS-CoV-2 virus were conducted in a BSL3 facility, in accordance with the biosafety guidelines of the Israel Institute for Biological Research (IIBR).

### 2.3. VSV-∆G-Spike Preparation

VSV-∆G-spike was developed and generated, as previously described [6], followed by additional passaging in Vero cells (WHO, Vero RCB 10–87), which were widely accepted by the WHO and regulatory agencies for the manufacturing of human viral vaccines, and they were further purified, formulated, and titered, as previously described [6].

### 2.4. Animal Experiments

All animal experiments, involving SARS-CoV-2, were conducted in a BSL3 facility. Animal studies were approved by the Israel Institute for Biological Research Institutional Animal Care and Use Committee (IACUC). Female K18-hACE2 transgenic mice (The Jackson Laboratory, Bar Harbor, ME, USA), being 6–8 weeks old, were maintained at 20–22 °C and a relative humidity of 50 ± 10% on a 12 h light/dark cycle. Animals were fed with commercial rodent chow (Koffolk Inc., Neot Hovav, Israel) and provided with tap water ad libitum. Mice were randomly assigned to experimental groups.

K18-hACE2 mice were vaccinated intramuscularly (i.m.) with 10^7^ pfu/mouse of VSV-ΔG-spike at a prime-boost (P + B) regimen during a three-week interval.

SARS-CoV-2 original virus, as well as alpha, beta, gamma, or delta variants, were diluted in phosphate-buffered saline (PBS), supplemented with 2% FBS (Biological Industries, Beit-Haemek, Israel). Approximately 4 weeks following boost vaccination, anesthetized animals (75 mg/kg ketamine, 7.5 mg/kg xylazine in PBS) were infected by (20 µL i.n. instillation) with a pre-determined lethal dose as follow: 40 LD_50_ of the original virus, or 1000 LD_50_ variants alpha, 30 LD_50_ of beta, 100 LD_50_ of gamma, or 100 LD_50_ of delta. Animals’ body weight was monitored daily. Animals were sacrificed at 2 and 5 dpi for the following analyses: (1) viral load in lungs (2 and 5 dpi), nasal turbinates (2 dpi), and brains (5 dpi); (2) histopathological analysis in lungs and brains (5 dpi); and (3) RNAseq analysis of brains (5 dpi). Viral load, histopathological analysis, and RNAseq analysis are described below. The numbers of animals per experiment are indicated for each experiment.

### 2.5. Plaque Reduction Neutralization Test

Vero E6 cells were seeded in 12-well plates (4 × 10^5^ cells/well) and grown overnight in growth medium. Sera from mice following prime and boost vaccination were heat inactivated (HI) (56 °C for 30 min), and then they were diluted in twofold serial dilutions (between 1:40 and 1:5120) in 300 µL of infection medium (MEM containing 2% FBS with NEAA, glutamine, and P/S/N), mixed with 300 µL of 300 pfu/mL of original SARS-CoV-2, or each variant, and incubated at 37 °C, 5% CO_2_ for 1 h. Then, Vero E6 cells were infected with virus–serum mixtures (200 µL/well) and incubated at 37 °C and 5% CO_2_ for 1 h. 2 mL per well overlay (MEM containing 2% FBS and 0.4% tragacanth (Merck, Jerusalem, Israel)) were added to each well, and plates were incubated at 37 °C 5% CO_2_ for 72 h for original virus, beta, gamma, and delta variants or 96 h for alpha variant. Following incubation, the overlay was aspirated, and cells were fixed and stained with 1 mL/well of crystal violet solution. The number of plaques in each well was determined, and the serum dilution that neutralizes 50% of the virions (NT_50_) was calculated using Prism software (GraphPad Software Inc.).

### 2.6. Viral Load Determination in Organs

Viral load was determined for nasal turbinates (2 dpi), lungs (2 and 5 dpi), and brains (5 dpi). Organs were harvested, and they were processed, and infectious virus quantitation was performed by plaque assay, as previously described [6]. Briefly, serial dilutions of extracted organs were prepared in infection medium (MEM containing 2% FCS) and used to infect Vero E6 monolayers in duplicates (200 µL/well). Plates were incubated for 1 h at 37 °C to allow viral adsorption. Then, 2 mL/well of overlay (MEM containing 2% FBS and 0.4% tragacanth (Merck, Jerusalem, Israel) was added to each well, and plates were incubated at 37 °C, 5% CO_2_, for 72 h (for original, beta, gamma, and delta variants) or 96 h (for alpha variant). The medium was then aspirated, and the cells were fixed and stained with 1 mL/well of crystal violet solution (Biological Industries, Beit-Haemek, Israel). The number of plaques in each well was determined. Viral load, as well as LOD, were calculated based on volume of cell infection (200 µL/well), dilution factor, and tissue processing volume (1 mL for nasal turbinates, 1.5 mL for lungs and brains), and they are presented as pfu/organ.

### 2.7. Histopathology and SARS-CoV-2 Immunohistochemical Staining

Virus detection was performed by immunohistochemistry (IHC), and mice (*n* = 3 per group) were anesthetized, and then they were perfused transcardially with PBS. Lungs and brains were isolated and fixed in 4% paraformaldehyde at room temperature (RT) for 7 days, and then they were transferred to 70% ethanol for further fixation. Lungs were spread horizontally in the cassette, and brains were dissected into four coronal sections, showing the striatum, hippocampus, midbrain, cerebellum, and brainstem. Serial sections, 4 µm thick, were performed using the Leica BOND-MAX system (Leica Biosystems Newcastle Ltd., Newcastle upon Tyne, UK). For 3, 30-diaminobenzidine (DAB) and H&E staining, pictures were taken using the Olympus microscope (BX60, Serial No. 7D04032, Tokyo, Japan) equipped with the microscope’s camera (Olympus DP73, Serial No. OH05504) at objective magnifications of 1.25× and 10×.

Paraffin sections (4 microns thick) were cut, put on glass slides, and stained for viral detection by IHC. Immunohistochemical staining was performed on 4 µm paraffin sections using the Leica Bond max system (Leica Biosystems Newcastle Ltd., Newcastle upon Tyne, UK). Sections were dewaxed and pretreated with epitope-retrieval solution for 10 min (ER1, Leica Biosystems Newcastle Ltd., Newcastle upon Tyne, UK), followed by 30 min incubation with a rabbit anti SARS-CoV-2 antibody (in-house preparation of rabbit polyclonal anti-RBD, diluted 1:800). Pictures were taken using an Olympus microscope (BX60, serial NO. 7D04032), equipped with a microscope camera (Olympus DP73, serial NO. OH05504) at objective magnification of ×1.25, ×10.

### 2.8. Gene Expression and Functional Analysis

RNA was isolated from mice brains at 5 dpi using Qiagen RNeasy mini kits (Qiagen, Valencia, CA, USA) with an on-column DNase step (Qiagen, Valencia, CA, USA), according to the manufacturers’ instructions. RNA quantification was carried out in a Qubit fluorometer using the Qubit RNA HS assay kit (Invitrogen, Carlsbad, CA, USA). Quality control analysis of RNA integrity was performed using High Sensitivity RNA ScreenTape and the TapeStation Analysis software (Agilent Technologies, Santa Clara, CA, USA). All the samples reached a RIN (RNA Integrity Number) score of >7. RNA-seq was performed at the JP Sulzberger Columbia Genome Center (New York, NY, USA). Libraries were generated using the Illumina TruSeq stranded mRNA kit, according to the manufacturer’s instructions. Polyadenylated RNA enrichment was performed. Sequencing of the 100-bp paired-end reads was performed on the Illumina NovaSeq 6000 system. DESeq2 files were constructed by a comparison of the data of the virally infected mice with the corresponding carrier buffer control of the same time point for log2 fold change and *p*-values. The log2 fold change was converted to fold change values in some cases for simplicity.

Functional analysis was performed by Ingenuity Pathway Analysis (IPA^®^; Qiagen Redwood City, CA, USA) to evaluate the influence of the viral infection on biological canonical pathways and networks. IPA cutoff criteria for input list of differentially expressed genes was set to log2 fold-change > ±1.3 and adjusted *p*-value < 0.05. Graphical representation of networks with their scores were computed by IPA. Within networks, nodes represent genes and lines indicate biological relationships (direct or indirect) with other genes, based on the published literature within the IPA software. The network score is based on hypergeometric distribution and is calculated with the right-tailed Fisher’s exact test.

### 2.9. Statistical Analysis

Data were analyzed with GraphPad Prism 6 software. Exact *p* values are provided for each analysis. Statistical significance for neutralization of SARS-CoV-2 variants was determined by one-way ANOVA, as well as the Kruskal-Wallis test with Dunns’ multiple comparisons test. Statistical analysis for mortality was performed using the Log-Rank (Mantel-Cox) test. Significance for viral load analysis was determined by the Mann-Whitney nonparametric test, per organ (vaccinated vs. unvaccinated). For IPA, the network score was based on hypergeometric distribution and was calculated with the right-tailed Fisher’s exact test.

## 3. Results

### 3.1. VSV-ΔG-Spike Vaccination of K18-hACE2 Mice Maintains Neutralizing Antibodies against Alpha, Beta, Gamma, and Delta Variants

**Figure 1:**
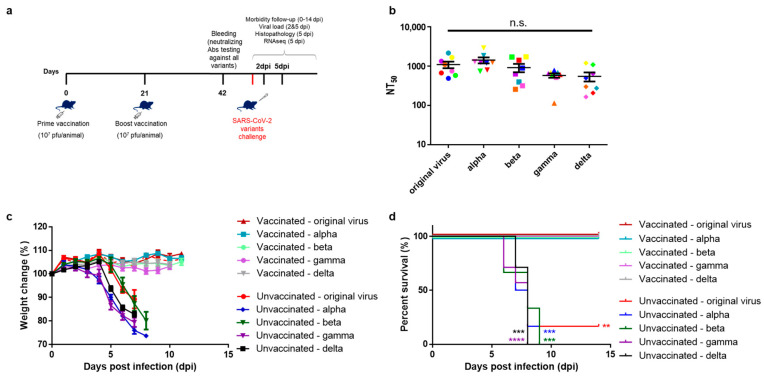
VSV–ΔG–spike induces antibodies capable of efficient neutralization of SARS-CoV-2 variants, and protects K18–hACE2 mice against lethal doses of SARS-CoV-2 variants. (**a**) Study design: K18–hACE2 mice were vaccinated i.m. with VSV-∆G-spike vaccine (10^7^ pfu, prime and boost with 3 weeks interval). Approximately 4 weeks following boost vaccination, mice were challenged intranasally (i.n.) with SARS-CoV-2 original virus (40 LD_50_), or variants alpha (1000 LD_50_), beta (30 LD_50_), gamma (100 LD_50_) or delta (100 LD_50_), to enable uniform lethality of the animals. Animals were monitored daily for morbidity and mortality, and subjected to further analyses. (**b**) Mice sera 3 weeks post boost vaccination were tested for neutralization of variants. NT_50_ values of VSV–ΔG–spike i.m. vaccinated K18–hACE2 mice sera (*n* = 8) showing neutralization of SARS-CoV-2 original virus, and alpha, beta, gamma and delta variants. Each color represents a serum sample. Data is presented as mean ± SEM. Statistical analysis was performed using one-way ANOVA, with Dunn’s multiple comparisons test. n.s. = not significant. (**c**) Morbidity (body weight changes shown as % of initial weight) of VSV-∆G-spike vaccinated K18–hACE2 mice infected with either the original SARS-CoV-2 virus (*n* = 7), alpha (*n* = 7), beta (*n* = 6), gamma (*n* = 9) or delta variants (*n* = 10), or unvaccinated mice: *n* = 6 for original virus, alpha and beta; *n* = 7 for gamma and delta variants. Dashed red line represents the 1 surviving mouse of the unvaccinated-original virus group. (**d**) Survival curve of vaccinated and unvaccinated K18–hACE2 mice, infected with either the original virus, alpha, beta, gamma or delta variants. Data is presented as mean ± SEM. Statistical analysis was performed using Log-Rank (Mantel-Cox) test, ** *p* < 0.01, *** *p* < 0.001, **** *p* < 0.0001.

First, we evaluated the induction of neutralizing antibodies against SARS-CoV-2 in VSV-ΔG-spike-vaccinated K18-hACE2 mice. To that end, K18-hACE2 mice were vaccinated intramuscularly (i.m.) with 10^7^ pfu/mouse of VSV-ΔG-spike at a prime-boost (P + B) regimen, in a three-week interval (Figure 1a). Three weeks following boost vaccination, mice sera were tested by 50% plaque reduction neutralization test (PRNT_50_) for the level of neutralizing antibodies against several authentic SARS-CoV-2 variants: the original virus, alpha, beta, gamma, or delta. All tested sera were able to efficiently neutralize all tested variants (Figure 1b). There was no significant difference between the neutralization capacity of mice sera against the original virus and the tested variants.

### 3.2. VSV-ΔG-Spike Vaccination Protects Mice against Lethal Challenge of SARS-CoV-2 Variants

For each variant, the lethal dose in unvaccinated mice was pre-determined. For the original virus, LD50 = 50 pfu, alpha LD50 = 0.5 pfu, beta LD50 = 300 pfu, gamma LD50 = 30 pfu, and delta LD50 = 100 pfu. Then, VSV-ΔG-spike-vaccinated mice were challenged intranasally (i.n.), accordingly, with a lethal dose of one of the following SARS-CoV-2 variants: original virus, alpha, beta, gamma, or delta, and they were monitored daily for body weight changes (Figure 1c) and survival (Figure 1d). All vaccinated mice did not display any weight loss following infection and throughout the experiment, and all survived the lethal challenge of either the original virus, or any of the variants’ challenge, whereas all of the unvaccinated and infected mice, except for one mouse infected with the original virus, succumbed to the disease at a similar rate.

### 3.3. VSV-ΔG-Spike Vaccination Reduces Viral Load in Target Organs

**Figure 2:**
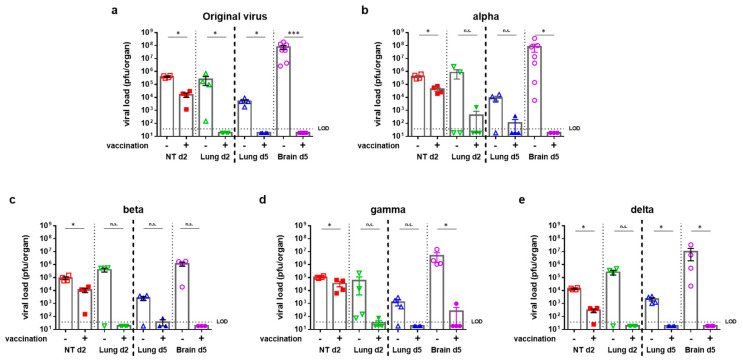
VSV–ΔG–Spike vaccination reduces viral load in organ tissues of K18–hACE2 mice infected with SARS-CoV-2 variant. Viral load analysis of unvaccinated K18–hACE2 mice or K18–hACE2 mice vaccinated prime and boost with 10^7^ pfu of VSV–∆G–spike, following i.n. infection with either (**a**) SARS-CoV-2 original virus, (**b**) alpha, (**c**) beta, (**d**) gamma, or (**e**) delta variants. Viral titers were tested at 2 dpi in nasal turbinates (NT) and lungs, and at 5 dpi in lungs and brains. Viral titers were determined by plaque assay, and are presented as pfu/organ. Data is presented as mean ± SEM. For each organ at each time point, *n* = 4; original virus and alpha infected brains at 5 dpi: *n* = 7; beta variant lungs and brains at 5 dpi: *n* = 3. Values below limit of detection (LOD) were assigned values of half LOD. Each color represents different organ. Statistical analysis was performed using Mann–Whitney nonparametric test, per organ (vaccinated vs. unvaccinated). * *p* < 0.05, *** *p* < 0.001, n.s. = not significant.

For viral load analyses, target organs were examined at several time points following SARS-CoV-2 variants’ infection; lungs and nasal turbinates were examined at two days post infection (dpi), and lungs and brains were examined at five dpi, and they were tested for the presence of live virus by plaque assay (Figure 2).

For all tested variants, infection of unvaccinated animals led to significant virus propagation in the nasal turbinates at day two post infection (Figure 2). As for the lungs, some animals displayed high viral titers at two dpi, whereas others showed lower or non-present values, regardless of the variant. On day five post infection, infected unvaccinated mice displayed high viral titers in the lungs (in the range of 10^3^ to 10^4^ pfu/lung) and very high titers in the brains (ranging between 10^5^ to 10^8^ pfu/brain, depending on the variant, Figure 2). VSV-ΔG-spike vaccination was able to reduce viral load in nasal turbinates at two dpi, and it was able to prevent viral load in the lungs at two dpi, and in both lungs and brains at five dpi, regardless of the specific variant used for infection; only a single vaccinated mouse infected with the gamma variant (Figure 2d) displayed residual (975 pfu) infectious virus in the brain at five dpi. Taken together, VSV-ΔG-spike vaccination significantly prevented viral load in target organs.

### 3.4. VSV-ΔG-Spike Vaccination Reduces SARS-CoV-2 Viral Antigen in Lung and Brain Tissues

**Figure 3:**
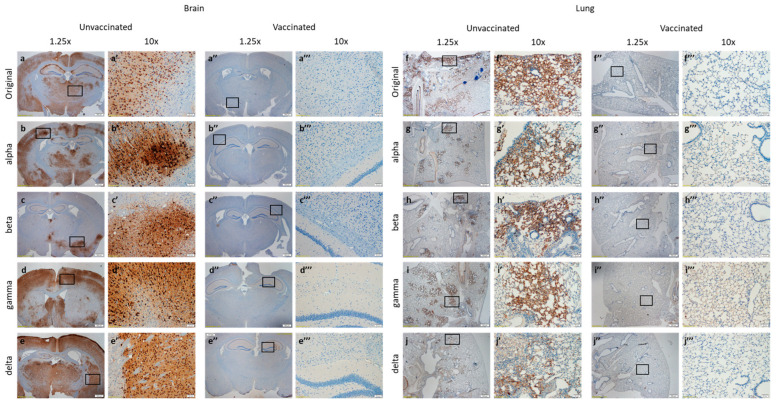
Immunohistochemical staining of lungs and brains of VSV-ΔG-spike vaccinated, or unvaccinated mice, following SARS-CoV-2 variants infection. Histopathological analysis showing immunohistochemical (IHC) staining of (**a**–**e**) brains and (**f**–**j**) lungs of unvaccinated ((**a**–**e**,**f**–**j**) low magnification, (**a’**–**e’**,**f’**–**j’**) high magnification) or vaccinated ((**a’’**–**e’’**,**f’’**–**j’’**) low magnification, (**a’’’**–**e’’’**,**f’’’**–**j’’’**) high magnification) at five dpi with either the original virus, alpha, beta, gamma or delta variants. Low magnification (×1.25) or insets at high (×10) magnification at approximate location are marked. Images are representative of *n* = 3 mice per group.

We next assessed the effect of vaccination on brain and lung pathology of K18-hACE2 infected with the original SARS-CoV-2 or variants alpha, beta, gamma, and delta, at the abovementioned lethal doses. Immunohistochemical (IHC) staining for SARS-CoV-2 antigens was performed on brains extracted at 5 dpi to evaluate the presence and tissue distribution of viral antigens. In brains of unvaccinated mice infected with SARS-CoV-2 or the tested variants (Figure 3a,a’,b,b’,c,c’,d,d’,e,e’), positive staining was visualized in all tested areas, namely, the striatum, hippocampus, midbrain, and cerebellum. Staining was pronounced in pyramidal neurons of the hippocampus and in specific neurons in other brain areas, such as the fontal cortex and septum. On the contrary, positive staining was not observed in vaccinated brains (Figure 3a’’,a’’’,b’’,b’’’,c’’,c’’’,d’’,d’’’,e’’,e’’’). IHC also showed positive staining in lungs of unvaccinated-infected animals, infected with any of the tested variant, as well as the original SARS-CoV-2 (Figure 3f,f’,g,g’,h,h’,i,i’,j,j’). Lungs of unvaccinated-infected animals displayed a broncho-interstitial viral pneumonia. The histological changes in lungs of unvaccinated mice infected with SARS-CoV-2 variants were almost identical to those described in the lungs infected with the original virus lungs, yet at different levels of severity. To the contrary, vaccination of the animals prior to infection led to a reduction in antigen presence in the infected lungs (Figure 3f’’,f’’’,g’’,g’’’,h’’,h’’’,i’’,i’’’,j’’,j’’’).

These data, showing marked reduction in positive SARS-CoV-2 staining in brains and lungs of vaccinated mice, correspond with viral load analysis in target organs, showing viral presence in the lungs of the unvaccinated and infected animals, as well as very high viral titers in the unvaccinated and infected brains at five dpi, in contrast to a nearly complete clearance of viral load from both lungs and brains in infected mice following vaccination.

### 3.5. SARS-CoV-2 Variants Lead to Significant Changes in Brain Gene Expression, Which Are Prevented by VSV-ΔG-Spike Vaccination

Thus far, we show VSV-ΔG-spike vaccine efficacy in prevention of disease manifestations, as well as reduction in viral load in target organs. To further analyze vaccine efficacy against variants’ challenge, we analyzed, by RNAseq, the gene expression profile in the brains of unvaccinated mice that were infected with the original virus, or alpha, beta, gamma, or delta variants. To achieve a comprehensive profile of genes and pathways affected by SARS-CoV-2 infection, we focused on the brain as a major target organ in the K18-hACE2 model. The expression profile at 5 dpi was compared between unvaccinated and vaccinated mice. To that end, two sets of comparisons were performed: the first set is defined herein as “set 1” or “unvaccinated and infected disease state” and is comprised of unvaccinated mice infected with either the original virus, alpha, beta, gamma, or delta. The second set includes VSV-ΔG-spike-vaccinated mice infected with the abovementioned variants, and it is termed “set 2” or “vaccinated”.

**Figure 4:**
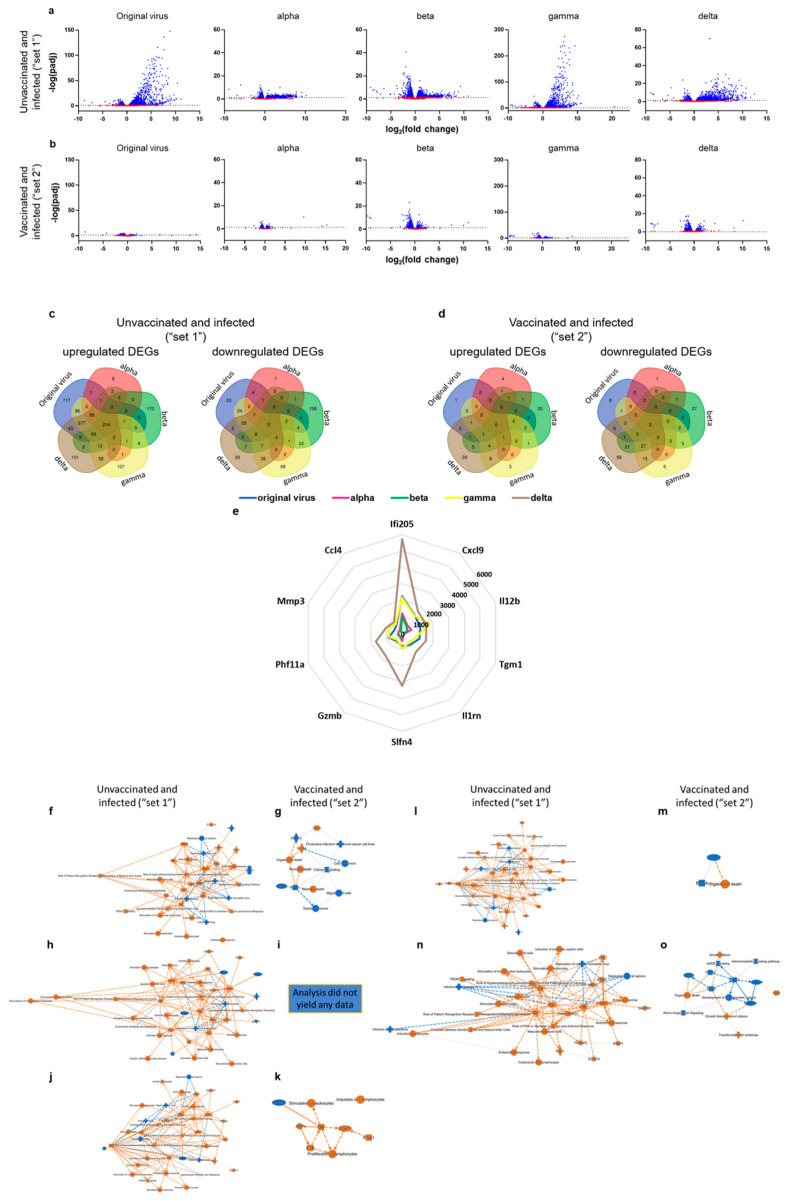
Distinct transcriptional signatures of brains of unvaccinated mice and VSV-∆G-spike vaccinated mice, following infection with any of the SARS-CoV-2 variants. RNA-seq analysis of brain homogenates of K18–hACE2 mice, unvaccinated and infected with SARS-CoV-2 variants (“set 1”), or vaccinated with VSV-∆G-spike and infected with SARS-CoV-2 variants (“set 2”), at 5 dpi. (**a**,**b**) Volcano plots showing differentially expressed genes (DEGs) of brains taken from (**a**) unvaccinated mice or (**b**) VSV-∆G-vaccinated mice 5 days following infection with original virus, alpha, beta, gamma or delta variants. Fold change (FC) >2 or <0.5 and false discovery rate (q value) < 0.05. (**c**,**d**) Venn diagrams of overlapping genes identified in differential expression analysis, showing (**c**) upregulated (left) or downregulated (right) genes of “set 1” for brains of unvaccinated mice infected with each variant compared to naïve mice, and (**d**) upregulated (left) or downregulated (right) genes of “set 2” for brains of VSV-∆G-spike-vaccinated mice infected with each variant compared to naïve mice. For **c,d**, number of genes are displayed. *n* = 3 for each group. (**e**) Radar chart highlighting the top 10 upregulated DEGs in unvaccinated mice brains 5 days following infection with the original virus and their FCs with alpha, beta, gamma or delta variants. (**f**–**o**) Graphical summary showing the major biological themes in the Ingenuity Pathway Analysis (IPA), illustrating the relationship of these themes to one another, for (**f**,**g**) original virus, (**h**,**i**) alpha, (**j**,**k**) beta, (**l**,**m**) gamma, (**n**,**o**) delta variants in the unvaccinated and infected and the vaccinated and infected brains, respectively. Selection is performed according to the most significant entities predicted in the analysis. *n* = 3 for each group. Orange nodes—predicted activation, blue nodes—predicted inhibition, lines represent relationship between nodes.

We first characterized changes in gene expression as a result of infection with each variant. At the disease state set, hundreds of genes were differentially expressed at each infected group, regardless of the specific variant, with the highest number of differentially expressed genes (DEGs) in the gamma-infected mice (Figure 4a,c). Venn analyses for upregulated or downregulated genes of all five variants for each set (“unvaccinated set” and “vaccinated set”) were performed to assess the extent in which these DEGs are shared by all variants, or rather unique. Most DEGs were upregulated (Figure 4c-left panel, fold change > 2). Gamma infection resulted in the highest number of upregulated genes (1042), most of which overlap with DEGs induced by infection with the other variants, as well. The beta variant displayed the most distinct unique DEGs pattern, with 170 upregulated genes that are specific to beta infection and do not overlap with any of the other variants. Alpha variant infection led to the smallest number of DEGs, both shared or unique to this specific variant, a total of 323 upregulated genes, and only 42 downregulated genes (fold change < 0.5). Altogether, 214 upregulated genes overlap between all tested variants in the unvaccinated set (Figure 4c-left panel), but only five downregulated genes overlap between the variants (Adgrl4, Ptprb, Slc22a8, Apcdd1, and Acta2, Figure 4c-right panel). Interestingly, for the original virus, as well as alpha, gamma, and delta variants, most genes were upregulated (~84–90% of the genes). However, for the beta variant, more than 30% of the DEGs were downregulated.

On the contrary, for all vaccinated and infected groups, only dozens of genes (for original, alpha, and gamma variants), or less than 200 genes (for beta and delta) were differentially expressed (Figure 4b,d, fold change > 2 or < 0.5, *p* adj < 0.05). Delta infection led to the highest number of DEGs in the vaccinated set (either upregulated or downregulated). Notably, for the vaccinated and infected set, there were no shared upregulated genes, and only two shared a downregulated gene (Ccm2l and St6galnac2). Graphical summaries comparing the overall major significantly affected biological entities for each variant following infection, without or following VSV-∆G-spike vaccination, were also created (Figure 4f,h,j,l,n compared to Figure 4g,i,k,m,o, respectively). We show that, whereas all unvaccinated mice brains display robust expression and activation of various genes and pathways (Figure 4f,h,j,l,n), vaccination with VSV-∆G-spike prior to infection completely prevented the observed biological activity, and it did not induce significant changes relative to the naïve mice brains (Figure 4g,i,k,m,o).

To further explore the disease state caused by the different variants, we also examined the most highly upregulated genes by the original virus, as well as their expression as a result from infection with any of the other variants—alpha, beta, gamma, or delta. The 10 top upregulated genes by the original virus (relative to naïve, sorted by the highest fold changes) include cytokines and chemokines (Cxcl9, Il12b, and Ccl4) and interferon activated or induced genes (Ifi205, Slfn4, Phf11a). Interestingly, these top upregulated genes are also ranked among the top upregulated genes in brains of mice infected by the SARS-CoV-2 variants, including alpha, gamma, and delta, and this was 8/10 for the beta variant (Table S1). Altogether, many of the highest upregulated genes are shared by all tested SARS-CoV-2 variants, further strengthening the similarity in the disease pathways between SARS-CoV-2 variants, which are all prevented by prior vaccination by the VSV-∆G-spike vaccine.

### 3.6. VSV-ΔG-Spike Vaccination Prevents Neuroinflammation and Brain Cytokine Storm Derived by SARS-CoV-2 Variants

Next, we aimed to explore pathways most affected by SARS-CoV-2 infection. To that end, we utilized the Ingenuity Pathway Analysis (IPA) tool to analyze the DEGs in each set. Pathway analysis for the original virus, as well as alpha, beta, gamma, and delta variants without prior vaccination, indicating disease state, are shown in Figure S1a–e, respectively.

**Figure 5:**
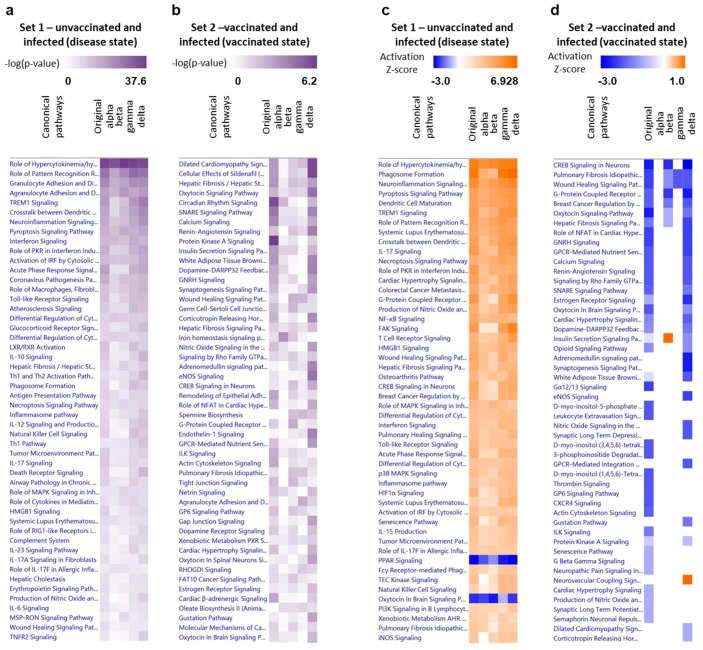
Comparison analysis of enriched and activated canonical pathways in brains of mice infected with SARS-CoV-2 variants. (**a**–**d**) Heat maps of comparison analyses summarizing the top 50 canonical pathways altered by each variant in the unvaccinated mice brains (**a**,**c**) or VSV-ΔG-spike vaccinated mice brains (**b**,**d**), analysed by Ingenuity Pathway Analysis (IPA). Pathways are ranked according to (**a**,**b**) −log(*p*-value), as well as according to (**c**,**d**) z-score, which predict activation (orange) or suppression (blue). Note the different scales for each comparison.

**Figure 6:**
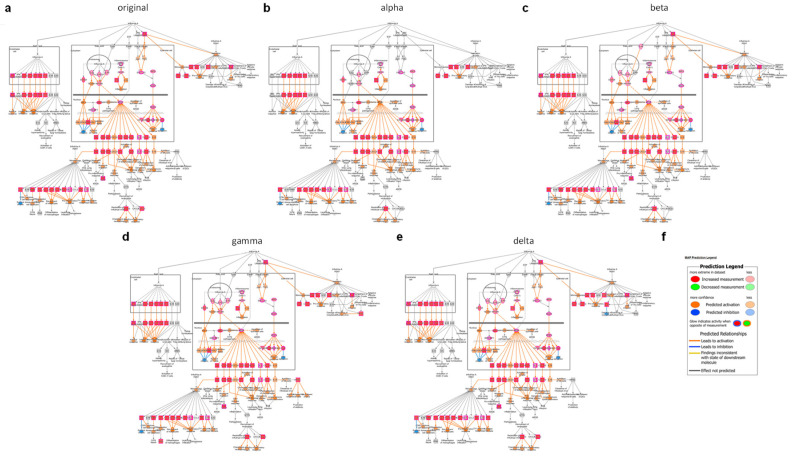
Top upregulated and activated canonical pathway “Role of Hypercytokinemia/hyperchemokinemia in the Pathogenesis of Influenza”. Upregulated expression is indicated by red nodes. Predicted activation is indicated by orange nodes. Lines indicate relations between molecules, whether direct (solid) or indirect (dashed). Schemes are presented for unvaccinated mice brains at five dpi with (**a**) the original virus, (**b**) alpha, (**c**) beta, (**d**) gamma, or (**e**) delta SARS-CoV-2 variant, showing a very similar activation patterns upon infection, regardless of SARS-CoV-2 variant identity. (**f**) Map legend.

We also performed comparison analysis of mice brains following infection with the original virus, as well as alpha, beta, gamma, or delta variants, with no prior immunity (Figure 5a,c) or following VSV-∆G-spike vaccination (Figure 5b,d). The top 50 common canonical pathways for each comparison are presented, ranked according to their fold change, as evident by −log(*p*-value) (Figure 5a,b), or according to their activation, as evident by z-score (Figure 5c,d). The top two pathways, according to −log(*p*-value), are “Role of Hypercytokinemia/hyperchemokinemia in the Pathogenesis of Influenza” (Figure 6) and “Role of Pattern Recognition Receptors in Recognition of Bacteria and Viruses” (PRR, Figure S2). Z-score, based comparison of canonical pathways of all variants in set 1, showed mostly upregulation of pathways for all variants, (Figure 5h), with the top three pathways being “Role of Hypercytokinemia/hyperchemokinemia in the Pathogenesis of Influenza” (Figure 6), “Phagosome formation” (Figure S3), and “Neuroinflammation Signaling Pathway”. (Figure S4). Among these 50 top pathways, only two are inhibited: “PPAR signaling” (#42) and “Oxytocin in brain signaling pathway” (#46).

Interestingly, and as opposed to set 1, a z-score analysis of the top canonical pathways of set 2, the vaccinated and infected brains, show mostly downregulation of the top 50 pathways, or no change relative to naïve animals, following infection with either original virus, alpha, beta, gamma, or delta variants (Figure 5d). Moreover, whereas the −log(*p*-value) and activation z-score scale bars are broad, (up to 37.6 for −log(*p*-value) and −3 to +6.928 for z-score), the z-score scale for vaccinated brains, following variant infection, is relatively narrow, ranging between −3 to +1, indicating that the fold change and activation in the vaccinated mice brains are insignificant, and the observed activation for set 2 is non-significant. Notably, infection with alpha variant, following vaccination, did not result in any significant pathway activation or inactivation.

Additionally, we compared the top five canonical pathways for each variant in the disease set (“set 1”), and in the vaccinated set (“set 2”), as shown in Table S2a. Pathway analysis of the disease set shows highly significant activation of common pathways, and each of them is represented by a substantial number of affected genes. On the contrary, the top five affected canonical pathway of the vaccinated set (“set 2”, Table S2b) differ between the five tested variants, and their −log(*p*-value) and the overlap of genes to each pathway are low, compared to the “set 1” values, altogether indicating a random assignment of the genes to these pathways.

Vaccinated mice brains do not display a significant host response to infection by any of the variants, indicating that the vaccine is able to prevent or control the disease caused by each variant. To that end, pathways expressed and activated by variants in the disease state represent the deleterious host response caused by SARS-CoV-2 infection, which can be avoided by prior immunization with VSV-ΔG-spike. We explored the #1 most expressed and activated pathway in all variants’ brains following infection in set 1, “Role of Hypercytokinemia/hyperchemokinemia in the Pathogenesis of Influenza”, and schemes detailing the involved components and their connectivity for each variant are shown (Figure 6a–e). For set 1, “unvaccinated disease state”, this pathway is characterized mostly by expression of a variety of cytokines, such as interleukins (ILs, i.e., IL1B, IL6, and IL10), interferons, tumor necrosis factor (TNF), and many others, and chemokines, as well as interferon-stimulating genes (ISGs) aimed at inducing an antiviral response indicated by orange pink and red shapes. A very similar expression and activation profile characterizes all of the tested variants in the disease state. Notably, IFNγ, a molecule involved in several arms of this pathway, is significantly increased only in gamma-infected mice brains. Additionally, IFNα is increased in all variants but alpha.

Altogether, these activated pathways, as well as others, are all aimed at inducing robust and elevated host immune response. However, the strong and excessive activation may result in an excessive proinflammatory cytokines activation, an imbalanced response, leading to hypercytokinemia or hyperchemokinemia. All of these were prevented by vaccination.

Taken together, VSV-ΔG-spike vaccination shows effective neutralization and protection, as well as prevention of deleterious disease manifestations that result from infection with all tested variants.

## 4. Discussion

Since the beginning of SARS-CoV-2 pandemic, emergence of new variants has been challenging the effectiveness of currently available vaccines, due to variants’ increased transmissibility’ and immune evasion [3,4,14]. Here, we explore the efficacy of VSV-ΔG-spike vaccine against the original SARS-CoV-2, as well as against several variants: alpha, beta, gamma, and delta in the K18-hACE2 mice model. We show complete protection of K18-hACE2 mice infected with SARS-CoV-2 variants, decrease in viral load and prevention of tissue damage by VSV-ΔG-spike vaccination. We also show that vaccination prevents brain-related cytokine storm exemplified by the robust transcriptomic disease profile, irrespective of variant identity.

Preclinical studies with our VSV-ΔG-spike vaccine candidate in four animal species (mice, rabbits, hamsters, and pigs) demonstrated its excellent safety profile [15,16]. Soon to be published data from the clinical trial in Israel, aiming to evaluate the safety, immunogenicity, and potential efficacy of VSV-ΔG-spike vaccine candidate (BriLife^®^) further indicates no significant adverse effects in humans.

Many have explored the ability of sera obtained from convalescent patients or human vaccinees to neutralize SARS-CoV-2 variants. Significant reduction in neutralization of the beta variant is often reported [3,4,8,9,17], whereas only a minor reduction in neutralization of alpha variant is described [2,4]. Neutralization of the delta variant is reduced by ~2–11 fold, which is more significant than the alpha, but less than the beta variant neutralization [4,13,18,19,20]. Here, we show significant neutralization of alpha, beta, gamma, and delta variants by vaccinated K18-hAEC2 mice sera with no significant difference in neutralization of the original virus. These data correspond with our previously reported results of sera samples from individuals receiving two-doses of VSV-∆G-spike as part of phase II clinical trial showing maintenance of the neutralization potential against several SARS-CoV-2 variants: alpha, beta, gamma, delta, and omicron, which were not tested in the current work [7]. During its development, rVSV-ΔG-spike vaccine spontaneously acquired mutations at specific sites, including, but not exclusive to, the receptor-binding domain (RBD). Some of them are identical to or correspond to key mutations or areas that can be found in several SARS-CoV-2 variants: N501, E484, Q493, and G685. These mutations are known to affect SARS-CoV-2 variants’ transmissibility and immune evasion. We suggest that these acquired mutations enable vaccine efficacy against the variants tested here, and potentially towards current and future SARS-CoV-2 variants.

With omicron and its subvariants being the predominant variants, we also aimed at establishing a robust infection model of K18-hACE2 with omicron variant. Infection of K18-hACE2 mice with omicron did not result in significant weight loss, nor led to lethality, even at high infection doses. This is in accordance with others reopting an attenuated disease in rodents, including K18-hACE2 mice, showing limited weight loss, if any, and a low viral burden in lower and upper respiratory tracts in K18-hACE2 mice [21], thus making the K18-hACE2 mouse model of low value in evaluating vaccine efficacy against Omicron. Hence, our work focuses on variants alpha, beta, gamma, and delta, presenting a lethal disease following infection.

Though the respiratory system is considered the major target of SARS-CoV-2, COVID-19 is a multi-organ disease, accompanied by central nervous system symptoms, such as memory and attention deficits, and neurological manifestations associated with severe prognosis [22]. ACE2 is expressed in human brains, mostly in neurons, but also in non-neuronal cells, such as astrocytes, oligodendrocytes, and endothelial cells, thus making the brain susceptible to SARS-CoV-2 infection [23,24]. Additionally, autopsies of post-mortem patients’ brain tissues revealed profound neuroinflammation phenotype, with substantial immune activation in the CNS, neuropathology, as evident by astrocytosis, axonal damage, blood–brain barrier leakage, and viral antigen presence in ACE2-receptor-positive cells enriched in the vascular compartment [25,26]. Recapitulating COVID-19 brain pathology in animal models is of major importance for assessing SARS-CoV-2 derived damage, as well as vaccine efficacy.

K18-hACE2 mice develop a robust respiratory disease resembling COVID-19 upon SARS-CoV-2 infection, with an emphasis on the brain as a major SARS-CoV-2 target. A recent examination of the spatiotemporal distribution and pathomorphological features in the CNS following intranasal infection with several SARS-CoV-2 variants showed that the original virus, as well as alpha, beta, and delta infection were restricted to neurons and appeared to spread from the olfactory bulb mainly in basally oriented regions in the brain and into the spinal cord, independent of ACE2 expression and without evidence of neuronal cell death, axonal damage, or demyelination [27]. The pathological damage to the lungs and brains of infected mice were recently found to be similar to the clinical symptoms of patients with severe COVID-19 disease, further suggesting that the K18-hACE2 model can recapitulate SARS-CoV-2 infection in humans [10]. In this work, we show that K18-hACE2 succumbed to infection with lethal doses of the original virus, as well as alpha, beta, gamma, and delta. Additionally, the SARS-CoV-2 tissue distribution depicted herein, for unvaccinated animals, is in accordance with other reports on K18-hACE2 mice model. It was previously reported that K18-hACE2 mice infected with 10^5^ pfu SARS-CoV-2 display the highest virus titers in lungs at three dpi, declining on days five and six dpi [11], and that disease severity, as well as death in K18-hACE2 mice, are correlated with peak SARS-CoV-2 viral load in the brain at five to six dpi [11]. Here, we show that VSV-ΔG-spike vaccination prevents all deleterious changes and effects, regardless of variant identity, in a similar manner. It was observed that there occurred prevention of weight loss and mortality, as well as elimination of virus presence, in lungs at two and five dpi and in brains at five dpi.

Viral load analysis showed high viral titers in unvaccinated mice brains, as well as histopathological evaluation, showing positive staining for SARS-CoV-2 in several brain areas in all brains. The robust VSV-ΔG-spike vaccination efficacy is further highlighted in these two analyses, where viral load is significantly reduced (below LOD), and viral antigen is not detected, irrespective of SARS-CoV-2 variant. In line with these findings, we also show a significant disease transcriptomic profile in the unvaccinated brains, representing disease state, as opposed to the vaccinated mice brains. Whether the lack of brain viral load, lack of viral antigen, and significant transcriptomic changes in vaccinated brain are derived from prevention of virus entry to the brain, or rather by its elimination, will require additional research, beyond the scope of this work. Nevertheless, thorough transcriptomic analysis of unvaccinated mice brains shed light on the extent and nature of brain disease caused by the different SARS-CoV-2 variants that are prevented by vaccination.

It was previously shown that early intranasal host response in the nasal epithelia, namely, site of infection, may underlie and precede disease severity [28]. This is mediated by interferon-stimulated genes (ISG). In mild and moderate COVID-19, there is an expression of anti-viral/interferon-responsive genes, which mediate the protective response, whereas severe disease is accompanied by impaired anti-viral gene expression on one hand, and recruitment of highly inflammatory macrophages, as the source of proinflammatory cytokines, on the other hand [28]. The significant reduction in virus titers we show in the nasal turbinates at two dpi in vaccinated mice may induce/correlate with ISG expression, leading to increased viral control, thus facilitating the lack of virus presence in the brain, altogether contributing to reduced disease severity and increased protection of K18-hACE2 mice. It is noteworthy that, whereas vaccination nearly eliminated virus presence in lungs and brains, in the nasal turbinates, at two dpi, there is a significant reduction in virus presence, but not complete exclusion. Hence, it would be interesting to explore intranasal administration of VSV-ΔG-spike, which may abolish viral presence, already, at the site of infection and reduce the risk of virus spread from infected individuals, thus providing an additional level of protection against SARS-CoV-2.

Due to the nature of the COVID-19 disease, and its impact on the respiratory system, many have explored gene expression patterns in lungs following SARS-CoV-2 infection. Winkler et al. [29] thoroughly analyzed lung inflammation and damage following SARS-CoV-2 infection with the original virus at several time points, showing high levels of viral RNA and viral titers at several time points following infection, decline in pulmonary function, as well as extensive upregulation of the innate immune response, including hundreds of DEGs, many of which associated with IFN signaling, nuclear factor-κB-dependent cytokine responses, or leukocyte activation [29]. Other work focused on RNAseq and bioinformatic data comparing transcriptional responses of mouse lungs (including K18-hACE2 mice), human lungs/lung tissues following SARS-CoV-2 infection showed differences between human and mice in single copy orthologues DEGs (scoDEGs), but high agreement in pathway analyses between species, specifically in immune signatures and inflammation pathways [30].

Here, we also aimed to characterize the transcriptomic landscape of brains infected with SARS-CoV-2 variants, representing diseased brains. We compared the diseased brain following infection with the original virus to infection with alpha, beta, gamma, or delta variants, and moreover, we tested the effect of VSV-∆G-spike vaccination prior to infection on the brain transcriptome. We show that diseased brains at five dpi upregulate hundreds of genes, whereas there are hardly any DEGs in vaccinated brains. Gamma and delta infection led to the highest number of upregulated DEGs in unvaccinated brains. There is a high proportion of upregulated DEGs that are shared by all variants. The top genes upregulated by the original SARS-CoV-2 infection are also among the 10–20 top genes upregulated by the other variants tested in this work: alpha, beta, gamma, and delta. The same applies for the top canonical pathways expressed by all variants in set 1, in which infection with any of the variants leads to significant activation of the same specific pathways: the first two pathways are shared by all tested variants: “Role of Hypercytokinemia/hyperchemokinemia in the Pathogenesis of Influenza” and “Role of Pattern Recognition Receptors in Recognition of Bacteria and Viruses”, followed by “Agranulocyte adhesion and diapedesis” and “Granulocyte adhesion and diapedesis”, pathways concerning with migration of leukocytes and white blood cells to areas of infection, and inflammatory response to pathogens, which are also highly ranked. Hypercytokinemia, or cytokine storm, and hyperchemokinemia accompanied by an uncontrolled proinflammatory response, play important roles in COVID-19 pathogenesis, and induction of pro-inflammatory cytokines was also correlated with disease severity in animal models and humans [12,31]. This is represented by the top expressed and activated pathway in our analysis. Though this pathway’s title highlights “…Pathogenesis of Influenza”, the molecules involved in this pathway are very much relevant for SARS-CoV-2, as well, including many cytokines and innate immune molecules, namely, IFNβ, IL1, IL6, IL10, Cxcl10, Cxcl2, IRF3, IRF7, IRF9, etc. SARS-CoV-2 infection is a potent inducer of proinflammatory cytokines, and as such, strong upregulation and activation of this pathway upon SARS-CoV-2 infection by any of the tested variants is expected [12,31].

The second highest-enriched canonical pathway by all variants in “unvaccinated set 1” is “Role of Pattern Recognition Receptors in Recognition of Bacteria and Viruses” (Figure S2). Pattern Recognition Receptors (PRRs) are important components of innate immunity, they recognize pathogen-associated molecular patterns (PAMPs), and this recognition leads to the expression of cytokines, chemokines, and co-stimulatory molecules, with the aim of eliminating infection and activating adaptive immunity [32]. SARS-CoV-2 was previously shown to be recognized by several distinct classes of PRRs: Toll-Like Receptors (TLRs), Retinoic-acid Inducible Gene-I Receptors (RLRs), and C-type Lectin Receptors (CLRs) [33]. Signaling through these PRRs leads to production of inflammatory cytokines and chemokines, as is also evident by the high activation of hypercytokinemia/hyperchemokiemia, as well as interferon signaling pathways.

In contrast, vaccination prior to infection with any of the tested variants leads to a relatively small amount of DEGs, mostly downregulated. For each variant in the vaccinated set, most DEGs (either upregulated or downregulated) were exclusive, with several genes overlapping between a few of the variants, and only two genes are shared by all variants. Such minimal overlap of DEGs between all vaccinated and infected mice with any of SARS-CoV-2 variants may imply that the vaccine prevents disease state by initiating a robust host response to the infection, such that the shared pathways and genetic signatures that were induced in the diseased animals are no longer activated. Additionally, canonical pathway expression and activation are less significant upon vaccination, as expressed by the relatively low *p*-value and z-scores. Thus, transcriptomic analysis highlights the ability of VSV-∆G-spike vaccination to prevent brain disease response caused by each of the tested variants in a similar manner.

Phagocytosis is an essential innate immune defense mechanism for the elimination of microbial pathogens. During viral infection, one important immune response is autophagy, which destroys the virus-infected cells, and viruses are often found to modulate it [34]. Here, “phagosome formation” is the second most activated canonical pathway shared by all tested variants (Figure S3). Formation is the first step of the three characteristic steps of phagocytosis: formation, maturation, and resolution [35]. The strong activation of this pathway indicates an immediate defense response against viral infection that activates immune and inflammatory response against each one of the tested variants. Such activation, as part of disease state in the brain, has deleterious consequences.

An additional interesting and highly activated pathway in brains of all unvaccinated and infected mice is the “Neuroinflammation signaling pathway” (Figure S4). Neuroinflammatory signaling plays a key role in maintaining CNS homeostasis, including destruction and removal of damaging agents and clearing injured neuronal tissue. However, uncontrolled inflammatory response can result in destruction of normal brain tissue.

When comparing the top 50 canonical pathways shared by all variants in the disease set to the top 50 canonical pathways common to all variants in the vaccinated and infected set, we show a pattern in which, in the “disease state”, all but one of the top 30 pathways are highly upregulated, and the trend is shared by all variants. “PPAR signaling” is the first pathway to be inactivated by all variants. The Peroxisome Proliferator-Activated Receptor (PPAR) family acts as a ligand-activated transcriptional regulator. PPARs are known to have an established immune modulatory role, thus their down regulation in disease state may abolish this activity, leading to exacerbated inflammatory damage. PPAR agonists were previously reported as effective adjuvants for COVID-19 vaccines by modifying immunogenetics [36].

On the contrary, analysis of vaccinated and infected canonical pathways yielded mostly downregulated pathways among the top 50, if any. Original virus and delta-infected mice were the most affected groups in “vaccinated and infected set 2”, with the largest number of canonical pathways. For all variants following vaccination, −log(*p*-value) and z-score values were significantly lower, with a maximum of 6.2 −log(*p*-value) and z-score ranging between −3 to 1, representing mostly inactivation or no effect on canonical pathways. Taken together, canonical pathway analysis shows that vaccinated mice brains, following SARS-CoV-2 vaccination, are very much similar to naïve mice brains, indicating prevention of disease as a result of vaccination.

Interestingly, despite the massive gene expression and activation of many canonical pathways, including pathways characterized by an excessive immune response in unvaccinated infected mice, these animals ultimately succumbed to the disease caused by any of the tested variants. Moreover, these deleterious disease manifestations were not at all evident at brains of vaccinated mice, further strengthening vaccine efficacy in disease prevention. Whether the immune response prevented virus entry to the brain awaits further analyses.

As previously reported, VSV-∆G-spike vaccine development was accompanied by spontaneous acquisition of mutations that are similar or identical to some of the key naturally occurring mutations in SARS-CoV-2 variants [7], which may be beneficial in VSV-∆G-spike efficacy against a variety of SARS-CoV-2 variants, and this may account for its robustness. The striking similarity in the disease transcriptomic landscape between the original virus in relation to alpha, beta, gamma, and delta variants, despite their differences in virulence and infectivity, may also account for variants that may emerge in the future. In this work, VSV-ΔG-spikes’ efficacy was evaluated against five different viruses and was able to prevent the deleterious infection outcomes, irrespective of variant identity, thus showing promising potential for VSV-ΔG-spike vaccine ability to also protect against omicron and its subvariants, as well as against future variants.

In light of the predominance of mRNA vaccines for SARS-CoV-2, we wish to point out several advantages of the VSV vaccination platform utilized by VSV-ΔG-spike. First, VSV-ΔG-spike has two mechanisms of action: the antigen (SARS-CoV-2 Spike) is presented on the surface of the virus particle, similarly to its presentation on the surface of SARS-CoV-2, and it is also expressed in vaccine-infected cells that further secrete it and/or present it to the immune system. In contrast, mRNA vaccines use only the latter. Second, the VSV-ΔG-spike may be applicable at various vaccination routes. The VSV-ΔG-spike vaccine is a live virus and the spike antigens presented on its surface mediate infection of target cells expressing hACE2. This enables utilization of this vaccine by variety of vaccination routes, such as i.m., i.n., or orally (p.o), to induce preferred local and systemic immune response.

Taken together, we suggest that VSV-∆G-spike provides robust protection against a variety of SARS-CoV-2 variants in a similar manner. Maintenance of neutralization potential against each variant, complete protection from morbidity and mortality, significant viral load reduction for some organs, and complete elimination for others, as well as “naïve-like” transcriptomic profile, regardless of variant identity, highlight the robustness of the VSV-∆G-vaccine, as well as its potential against future SARS-CoV-2 variants.

## Data Availability

Data are contained within the article or Supplementary Material.

## Supplementary Materials

The following supporting information can be downloaded at: https://www.mdpi.com/article/10.3390/v15061364/s1. Table S1: Top 20 DEGs in unvaccinated and infected mice brains (“set 1”); Table S2: Top 5 canonical pathways in brains of unvaccinated and infected mice (“set 1”), and vaccinated and infected mice (“set 2”); Figure S1: Enrichment of canonical pathways in brains of mice infected with SARS-CoV-2 variants; Figure S2: “Role of Pattern Recognition Receptors in Recognition of Bacteria and Viruses”; Figure S3: “Phagosome formation”; Figure S4: “Neuroinflammation Signaling Pathway”.
