# Supplementary material for: VSV-ΔG-Spike Candidate Vaccine Induces Protective Immunity and Protects K18-hACE2 Mice against SARS-CoV-2 Variants
Source: Viruses. 2023 Jun 13;15(6):1364. doi: 10.3390/v15061364 (PMC10303402; doi:10.3390/v15061364)
Supplement: Supplementary file 1 [file viruses-15-01364-s001.zip › viruses-2423820-supplementary.pdf]

<u>Supplementary data:</u>

	Original virus		Alpha		Beta		Gamma		delta	
		FC		FC		FC		FC		FC
Top 10	Ifi205	2245	Ifi205	1197	Gm10925	1994	Gm14648	1618316	Ifi205	5726
	Cxcl9	1320	Il12b	594	Ifi205	1024	Ifi205	2166	Slfn4	3221
	Il12b	1173	Cxcl9	559	RSP13-Ps2	651	Il12b	1761	Gzmb	1718
	Tgm1	1120	Slfn4	503	Cxcl9	471	Cxcl9	1290	Phf11a	1683
	Il1rn	874	Gzmb	234	Il12b	354	Slfn4	953	Cxcl9	1650
	Phf11a	860	Phf11a	233	Slfn4	306	Tgm1	902	Acod1	1555
	Slfn4	817	BC023105	232	Phf11a	280	Mmp3	878	Il12b	1554
	Gzmb	591	Cd69	230	Gm112918	274	Phf11a	829	Tgm1	1537
	Ccl4	571	Mmp3	228	Gzmb	248	Il1rn	751	Il1rn	1451
	Mmp3	496	Cxcl10	216	Cd69	248	Ccl4	640	Clec4e	1402
Top 11-20	LT629149.4	471	Ccl4	214	Clec4e	183	Cxcl10	614	Cd69	1101
	Cxcl10	464	Tnf	208	Plac8	167	Gzmb	508	Mmp3	1032
	Ifi206	460	Ifi206	201	Tnf	167	Acod1	496	Plac8	995
	Cd69	456	Ccl12	197	Ifi206	141	Tnf	471	Cfb	899
	Bcl2a1d	391	Ccl2	190	Cxcl10	1341	Ifi206	462	Tnf	890
	BC23105	381	Il1rn	185	Ccl4	126	Cd69	455	Ifi206	880
	Ccl12	378	Acod1	172	Ccl12	124	Ccl2	437	Ccl4	802
	Slc10a6	373	Tgm1	144	Tgm1	120	Ccl12	400	Ly6i	668
	Gm49339	347	Plac8	134	BC23105	120	Lcn2	388	Cxcl10	632
	Lcn2	337	Ccl3	131	GM9797	112	BC023105	380	Prl1	581

Supplementary Table S1: Top 20 DEGs in unvaccinated and infected mice brains ("set 1"): (a) Top 10 DEGs, and (b) 11-20 top DEGs ranked according to fold change (FC) effected by infection with each of the tested variants. Top 10 genes of the original virus are colored to highlight their position in the top 20 genes upregulated by the other variants as well. Genes is black are shared by more than one variant. Gray genes are only in the top 20 of one of the variants.

a

unvaccinated and infected ("set 1")														
Original virus			Alpha			Beta			Gamma			delta		
	p-value	overlap		p-value	overlap		p-value	overlap		p-value	overlap		p-value	overlap
Role of Hypercytokinemia/hyperchemokinemia in the Pathogenesis of Influenza	2.30E-36	40.7 % 35/86	Role of Hypercytokinemia/hyperchemokinemia in the Pathogenesis of Influenza	1.5E-34	31.4 % 27/86	Role of Hypercytokinemia/hyperchemokinemia in the Pathogenesis of Influenza	2.4E-38	36.0 % 31/86	Role of Hypercytokinemia/hyperchemokinemia in the Pathogenesis of Influenza	6.6E-37	41.9 % 36/86	Role of Hypercytokinemia/hyperchemokinemia in the Pathogenesis of Influenza	3.08E-36	41.9 % 36/86
Role of Pattern Recognition Receptors in Recognition of Bacteria and Viruses	1.52E-28	23.7 % 37/156	Role of Pattern Recognition Receptors in Recognition of Bacteria and Viruses	9.5E-19	13.5 % 21/156	Role of Pattern Recognition Receptors in Recognition of Bacteria and Viruses	5.7E-24	17.3 % 27/156	Role of Pattern Recognition Receptors in Recognition of Bacteria and Viruses	6.5E-30	25.0 % 39/156	Role of Pattern Recognition Receptors in Recognition of Bacteria and Viruses	4.84E-28	24.4 % 38/156
Granulocyte Adhesion and Diapedesis	2.89E-24	19.0 % 36/189	Granulocyte Adhesion and Diapedesis	5.3E-17	11.1 % 21/189	Agranulocyte Adhesion and Diapedesis	2.3E-21	13.1 % 28/214	Granulocyte Adhesion and Diapedesis	2.6E-23	19.0 % 36/189	TREM1 Signaling	2.20E-26	36.4 % 28/77
TREM1 Signaling	1.06E-20	29.9 % 23/77	Agranulocyte Adhesion and Diapedesis	6.8E-16	9.8 % 21/214	Granulocyte Adhesion and Diapedesis	2.6E-19	13.2 % 25/189	TREM1 Signaling	2.2E-21	31.2 % 24/77	Crosstalk between Dendritic Cells and Natural Killer Cells	4.65E-24	30.8 % 28/91
Agranulocyte Adhesion and Diapedesis	2.30E-19	15.4 % 33/214	Interferon Signaling	1.4E-14	30.6 % 11/36	Pyroptosis Signaling Pathway	2.8E-17	19.4 % 18/93	Crosstalk between Dendritic Cells and Natural Killer Cells	1.8E-19	26.4 % 24/91	Granulocyte Adhesion and Diapedesis	9.99E-24	19.6 % 37/189

b

vaccinated and infected ("set 2")														
Original virus			Alpha			Beta			Gamma			delta		
	p-value	overlap		p-value	overlap		p-value	overlap		p-value	overlap		p-value	overlap
Protein Kinase A Signaling	6.70E-07	3.4 % 14/409	Iron Homeostasis Signaling Pathway	2.6E-04	3.6 % 5/139	G-Protein Coupled Receptor Signaling	1.8E-03	1.3 % 9/703	Agranulocyte Adhesion and Diapedesis	2.3E-03	1.9 % 4/214	Dilated Cardiomyopathy Signaling Pathway	1.56E-06	5.4 % 8/148
Circadian Rhythm Signaling	2.02E-06	4.1 % 11/269	Pyridoxal 5'-phosphate Salvage Pathway	2.5E-03	4.5 % 3/66	Inflammasome pathway	2.8E-03	10 % 2/20	Wound Healing Signaling Pathway	4.2E-03	1.6 % 4/252	Cellular Effects of Sildenafil (Viagra)	1.72E-06	5.3 % 8/150
SNARE Signaling Pathway	3.64E-05	5.1 % 7/136	Circadian Rhythm Signaling	4.9E-03	1.9 % 5/269	Wound Healing Signaling Pathway	3.2E-03	2.0 % 5/252	Spermine Biosynthesis	5.2E-03	50.0 % 1/2	Oxytocin Signaling Pathway	2.51E-05	3.2 % 9/282
Calcium Signaling	1.11E-04	3.7 % 8/218	Salvage Pathways of Pyrimidine Ribonucleotides	7.5E-03	3.1 % 3/98	HOTAIR Regulatory Pathway	4.0E-03	2.5 % 4/163	Th2 Pathway	5.5E-03	2.2 % 3/137	eNOS Signaling	2.72E-05	4.4 % 7/159
White Adipose Tissue Browning Pathway	3.32E-04	4.3 % 6/138	Sumoylation Pathway	8.6E-03	2.9 % 3/103	Germ Cell-Sertoli Cell Junction Signaling	4.7E-03	2.3 % 4/171	Dilated Cardiomyopathy Signaling Pathway	6.8E-03	2.0 % 3/148	Renin-Angiotensin Signaling	5.62E-05	4.9 % 6/122

Supplementary Table S2: Top 5 canonical pathways in brains of unvaccinated and infected mice (“set 1”), and vaccinated and infected mice (“set 2”): Top 5 canonical pathways in (a) brains of unvaccinated and infected mice (“set 1”), and (b) brains of vaccinated and infected mice (“set 2”). For each pathway, p-value is indicated, as well as overlap in percentage, and number of genes that participate in the pathway. The top 5 canonical pathways of the original virus are colored, to highlight their position among the Top 5 pathways of the other tested variants.

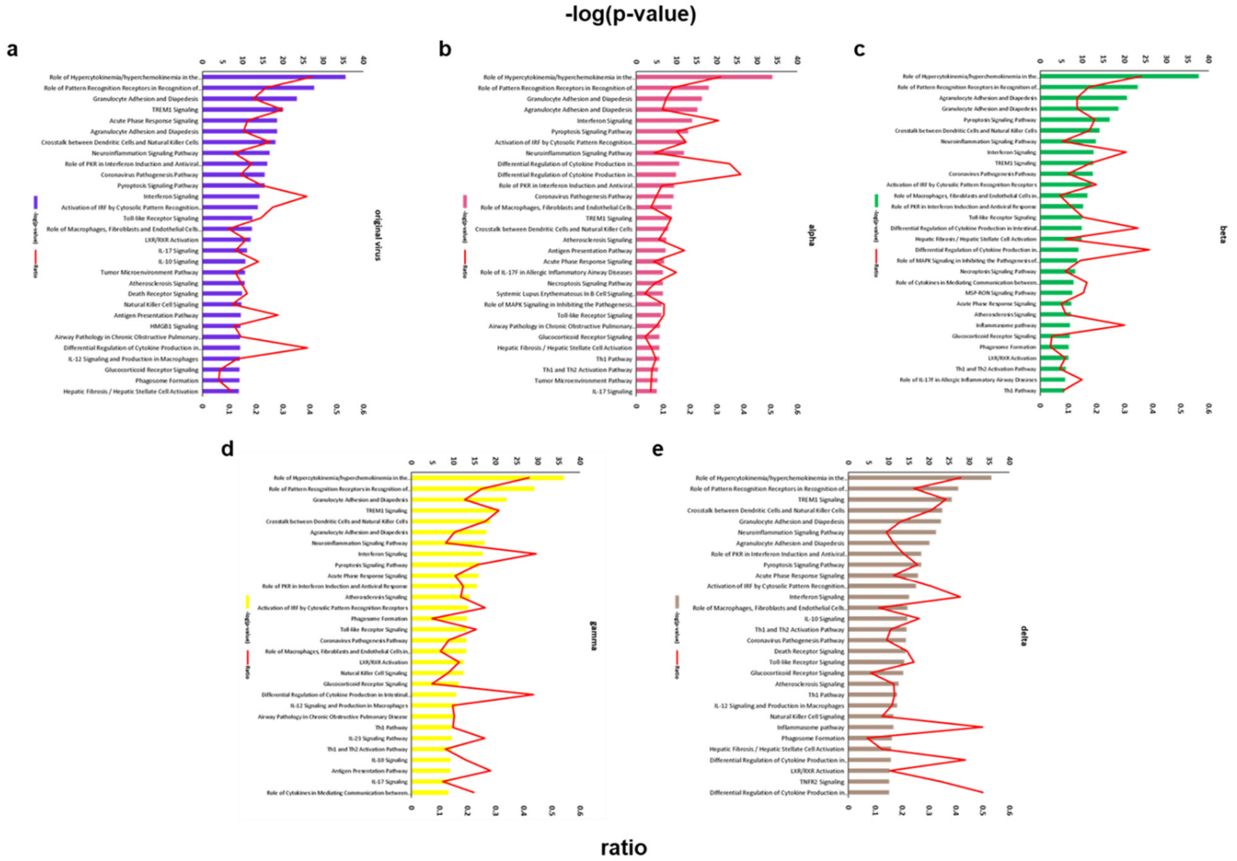

Supplementary Figure S1: Enrichment of canonical pathways in brains of mice infected with SARS-CoV-2 variants: Ingenuity pathway Analysis (IPA) of canonical pathways showing both upregulated and downregulated pathways ranked according to their −log (p-value) for each variant in "set 1" – unvaccinated mice infected with (a) the original virus (purple bars), (b) alpha (red bars), (c) beta (green bars), (d) gamma (yellow bars) or delta (brown bars). Analysis threshold is –log(p-value) >2. "Ratio" (red curve) indicates the number of molecules from the data set that map to the pathway listed divided by the total number of molecules that map to the canonical pathway from within the IPA database. X axis: pathways, left Y axis: -log(p-value), right Y axis: ratio.

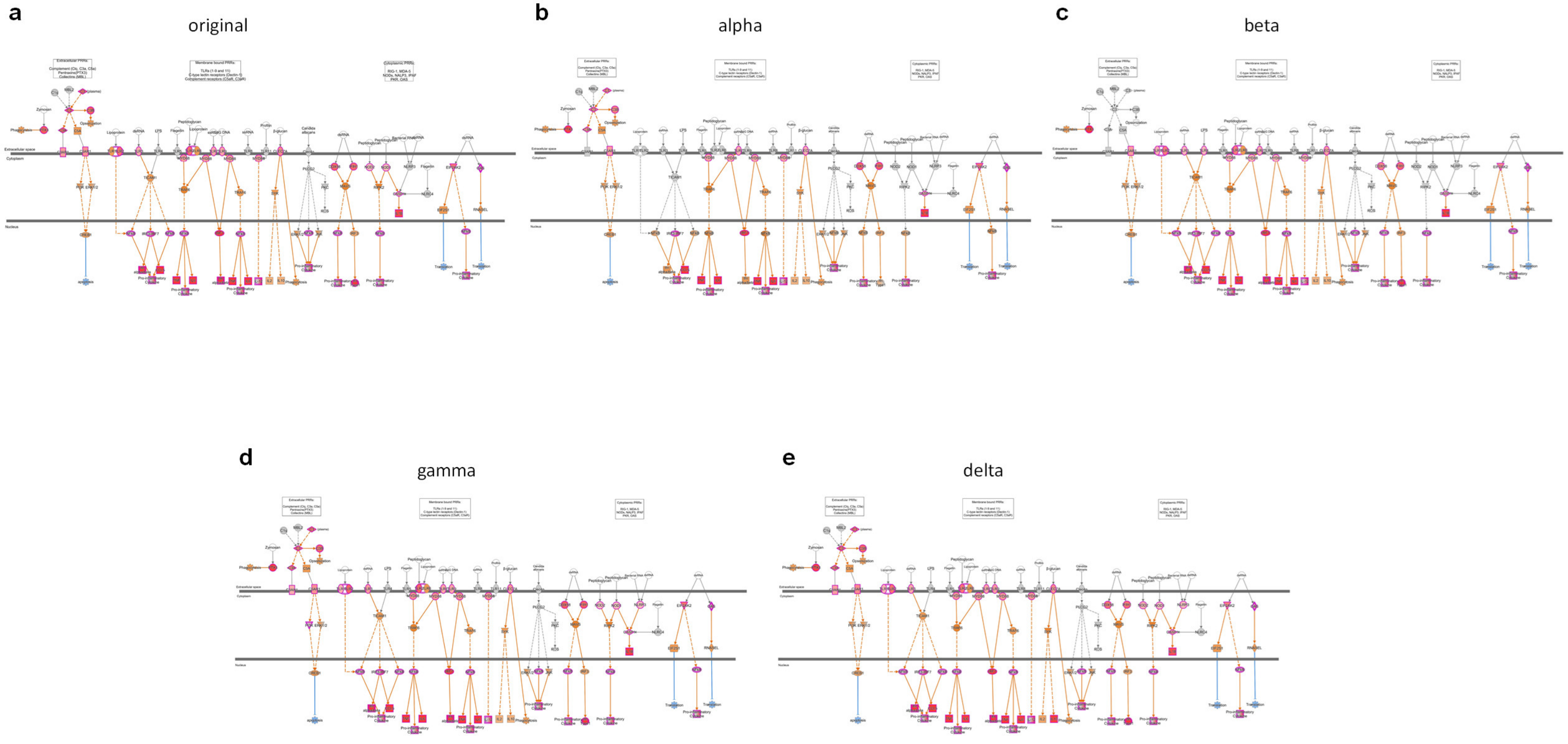

Supplementary Figure S2: “Role of Pattern Recognition Receptors in Recognition of Bacteria and Viruses” (PRR): Upregulated expression is indicated by red nodes. Predicted activation is indicated by orange nodes. Lines indicate relations between molecules wither direct (solid) or indirect (dashed). Schemes are presented for unvaccinated mice brains at 5 dpi with (a) the original virus, (b) alpha, (c) beta, (d) gamma or (e) delta SARS-CoV-2 variant, showing a very similar and activation patterns upon infection regardless of SARS-CoV-2 variant identity.

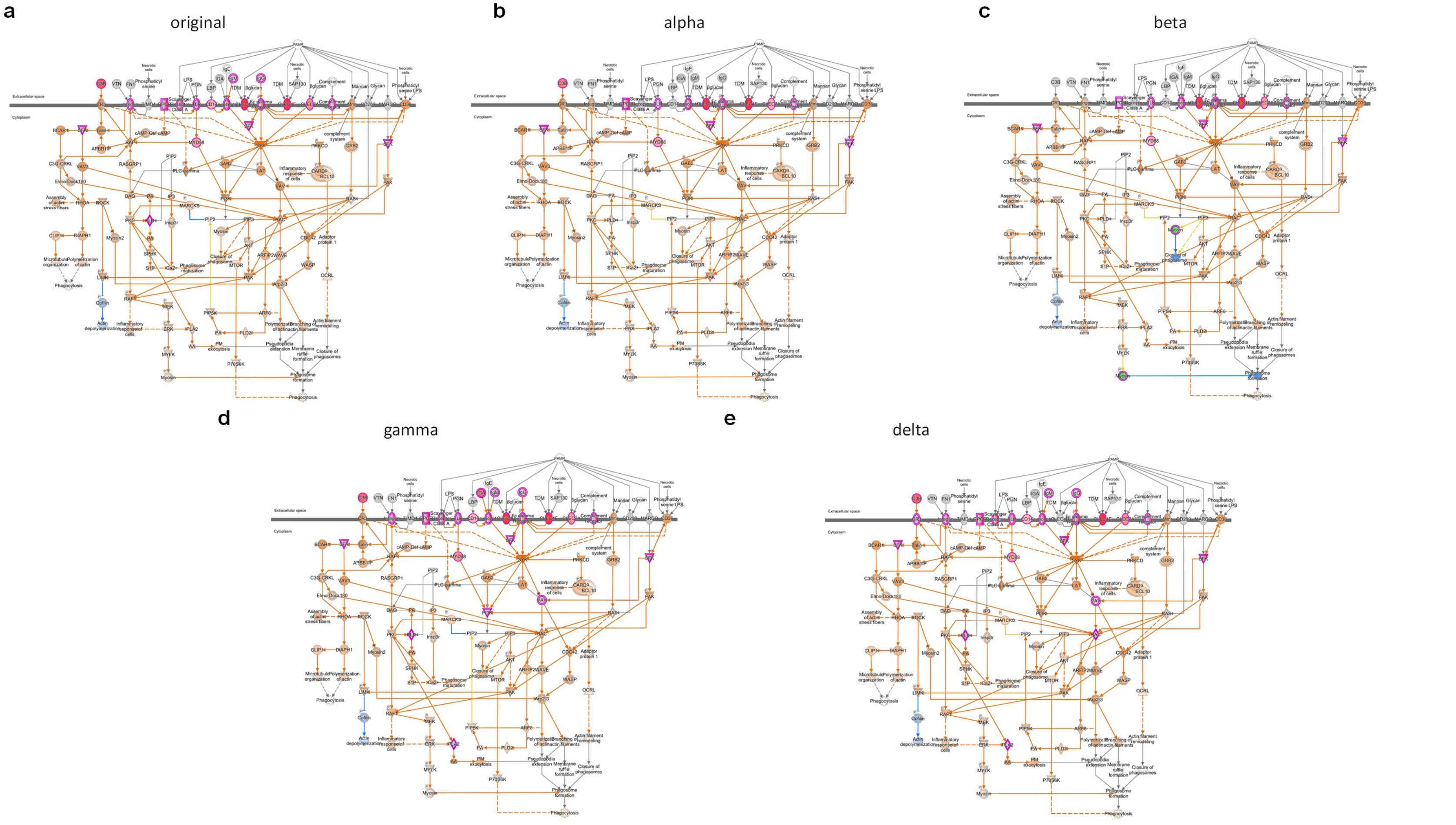

Supplementary Figure S3: "Phagosome formation": Upregulated expression is indicated by red nodes. Predicted activation is indicated by orange nodes. Lines indicate relations between molecules wither direct (solid) or indirect (dashed). Schemes are presented for unvaccinated mice brains at 5 dpi with (a) the original virus, (b) alpha, (c) beta, (d) gamma or (e) delta SARS-CoV-2 variant, showing a very similar and activation patterns upon infection regardless of SARS-CoV-2 variant identity.

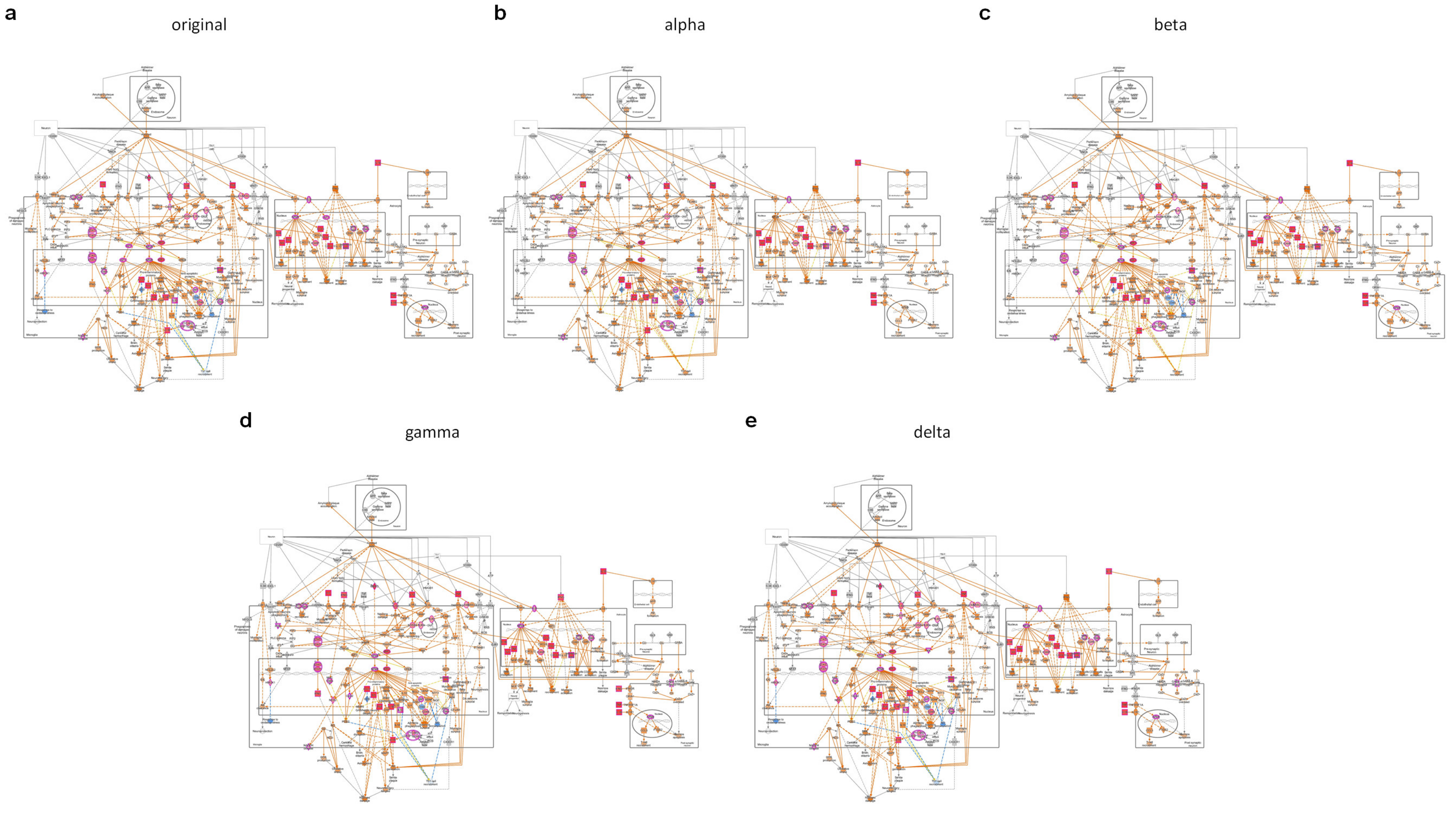

Supplementary Figure S4: “Neuroinflammation Signaling Pathway”: Upregulated expression is indicated by red nodes. Predicted activation is indicated by orange nodes. Lines indicate relations between molecules whether direct (solid) or indirect (dashed). Schemes are presented for unvaccinated mice brains at 5 dpi with (a) the original virus, (b) alpha, (c) beta, (d) gamma or (e) delta SARS-CoV-2 variant, showing a very similar and activation patterns upon infection regardless of SARS-CoV-2 variant identity.
